# Clinching of Thermoplastic Composites and Metals—A Comparison of Three Novel Joining Technologies

**DOI:** 10.3390/ma14092286

**Published:** 2021-04-28

**Authors:** Benjamin Gröger, Juliane Troschitz, Julian Vorderbrüggen, Christian Vogel, Robert Kupfer, Gerson Meschut, Maik Gude

**Affiliations:** 1 Institute of Lightweight Engineering and Polymer Technology, Technische Universität Dresden, Holbeinstraße 3, 01307 Dresden, Germany; juliane.troschitz@tu-dresden.de (J.T.); christian.vogel@tu-dresden.de (C.V.); robert.kupfer@tu-dresden.de (R.K.); maik.gude@tu-dresden.de (M.G.); 2 Laboratory for Material and Joining Technology (LWF), Paderborn University, Pohlweg 47-49, 33098 Paderborn, Germany; julian.vorderbrueggen@lwf.upb.de (J.V.); gerson.meschut@lwf.upb.de (G.M.)

**Keywords:** joining, clinching, thermoplastic composite, hybrid joint, multi-material design

## Abstract

Clinching continuous fibre reinforced thermoplastic composites and metals is challenging due to the low ductility of the composite material. Therefore, a number of novel clinching technologies has been developed specifically for these material combinations. A systematic overview of these advanced clinching methods is given in the present paper. With a focus on process design, three selected clinching methods suitable for different joining tasks are described in detail. The clinching processes including equipment and tools, observed process phenomena and the resultant material structure are compared. Process phenomena during joining are explained in general and compared using computed tomography and micrograph images for each process. In addition the load bearing behaviour and the corresponding failure mechanisms are investigated by means of single-lap shear tests. Finally, the new joining technologies are discussed regarding application relevant criteria.

## 1. Introduction

New legal requirements for reducing the carbon dioxide emissions of motor vehicles as well as customers’ increasing sensitivity for sustainability lead to the demand to reduce the total vehicle mass. This can be achieved, for example, by reducing the mass of the body-in-white structure using advanced lightweight designs. Due to their excellent specific mechanical properties and increasingly efficient manufacturing processes, thermoplastic composites (TPC) are an integral part of modern lightweight design concepts [1]. However, one challenge for the use of TPC in complex multi-material systems so far has been the availability of suitable joining systems. Mechanical joining processes like clinching are generally suitable for this purpose, as high lightweight degrees can be achieved and the costs of a single joining point can be minimised [2]. Furthermore, regarding the energy costs for the joining process, clinching shows advantages, especially over resistance spot welding [3]. The process is described in German standard DIN 8593-5 as the state of the art for joining by plastic forming. The process is used in automotive body-in-white production, the manufacture of electrical appliances, and coated components.

**Figure 1:**
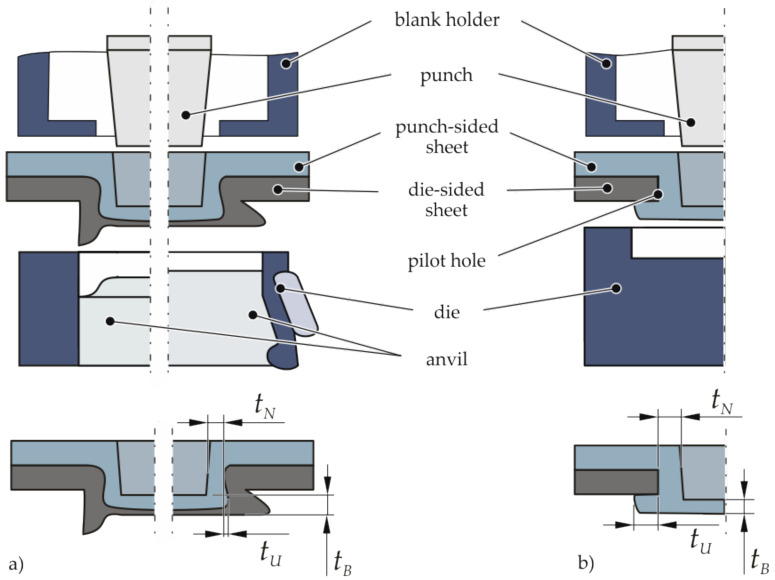
Clinching setup for (**a**) clinching with rigid or radial opening die and (**b**) clinching with pilot hole and relevant dimensions of clinching joints: neck thickness $t_{N}$, undercut $t_{U}$, and bottom thickness $t_{B}$.

The general clinchability of a joint depends on the ductility and the tensile strength of the joining partners. For the different requirements, various tools have been established [4]. In Figure 1, exemplary clinching tools and characteristic joint dimensions for clinching with rigid die (a-left), radial opening die (a-right), and clinching with pilot hole (b) are illustrated. If both joining partners are clinchable, rigid or radial opening dies can be used. For the clinching of materials with low ductility or high tensile strength, a pilot hole can be integrated in the less ductile joining partner, which has to be positioned on the die side [5].

**Figure 2:**
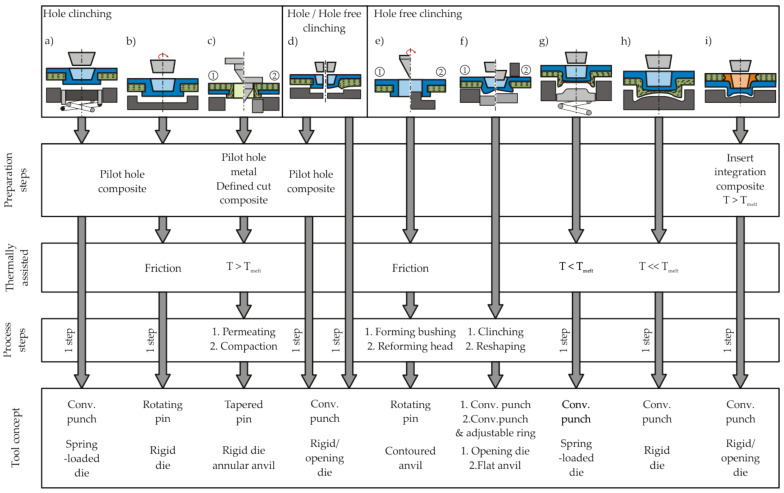
Classification of clinching processes: (**a**) Improved hole clinching [8], (**b**) Friction assisted Clinching [9], (**c**) Thermoclinching [16], (**d**) hole [6]/hole free [11,12] clinching, (**e**) Flow Drill Joining [15], (**f**) Two-Step Clinching [13], (**g**) Hotclinching [17], (**h**) Preheated Clinching [14], (**i**) Insert Clinching.

Due to the reduced ductility of TPC as well as restrictions from the endless fibre reinforcement, classical clinching is challenging for hybrid TPC-metal joints. Current research efforts are taking a variety of approaches to integrate TPC into multi-material structures using advanced clinching processes. Lee et al. describe a hole-clinching process (cf. Figure 2d right) for TPC using a rigid die. Due to dragging of the composite during the joining process, delamination occurred, which can reduce the load-bearing capacity of the joints [6]. They found that with increasing punch diameter, the neck thickness decreases, and the undercut increases [7]. Using a spring-loaded die (cf. Figure 2a), damage in the laminate can be reduced, as shown by Lee et al. [8]. In their work, the process window of joinable material thickness combinations was increased, since the spring-loaded anvil induces a hydrostatic compressive stress in the bottom area of the joint. This leads to less dragging of the laminate and reduced damage such as neck fractures in the punch-sided sheet during the joining process [8]. In order to increase the formability of aluminium joining partner during hole clinching with carbon fibre reinforced plastic (CFRP) with a thermoset matrix, Lambiase and Paoletti presented a process called friction-assisted clinching (cf. Figure 2b). A rotating tool is used heating up the punch-sided aluminium sheet to approximately 300 ${}^{\circ }$C. This enables crack-free joints with large undercuts even for low metal sheet thicknesses. The joining forces are significantly reduced due to the softening of the material [9].

Lambiase et al. also evaluated the clinching of polymer-metal joints and composite-metal joints without pilot hole (cf. Figure 2d left). For joints with aluminium and Polycarbonate [10], glass fibre reinforced plastic (GFRP) with a thermoset matrix [11] as well as CFRP [12], they showed that an undercut can also be achieved with conventional radial opening tools. Nevertheless, significant damage occurs in the composite, and the tools were partly contaminated by crumbled composite fragments, due to the brittleness of the TPC [12]. Additional compressive reshaping of the joint after clinching enlarged the undercut and the neck thickness (cf. Figure 2f). Nevertheless, an excessive reshaping of the joint can lead to a further increase in fibre buckling and delamination in the composite joining partner [13].

To reduce process induced damage, the ductility of TPC can be increased by heating the thermoplastic matrix. The promising approach of increasing the formability of TPC by process-integrated heating has been taken up by several authors. Lin et al. were able to observe the positive effect of heating the TPC when clinching Al-CFRP joints using a rigid die (cf. Figure 2h) [14].

Seidlitz et al. pursued a different approach using heat-based softening of TPC for a load-adjusted joining of multi-material structures without a pilot hole (cf. Figure 2e). In the Flow Drill Joining process, a metal-TPC joint is penetrated by a rotating metallic tapered pin creating a sleeve in the metallic joining partner and plasticising the TPC. This allows the realignment of the endless fibre reinforcement when the sleeve is formed without fibre breaking. Subsequently, a closing head is manufactured by a forming tool, creating an undercut [15].

It was pointed out that for TPC, process-integrated heating of the polymer matrix leads to a significant increase in formability. For this reason, three different technology approaches using thermally supported forming of TPC are discussed in more detail in this work: Thermoclinching (cf. Figure 2c), Hotclinching (cf. Figure 2g), and Insert Clinching (cf. Figure 2i). In addition to the design of the joining tools, the process cycle, and the joint geometry, the three technologies differ in terms of the joining direction. While the joining direction of Thermoclinching is TPC to metal, for Hotclinching, it is reversed. In contrast, the Insert Clinching is suitable for both directions. The phenomena occurring during the forming of the heated TPC as well, as the joint formation and performance for the three technologies, are compared in the following.

## 2. Process

### 2.1. Thermochlinching

**Figure 3:**
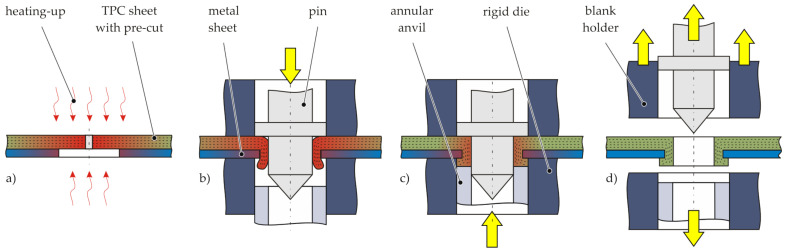
Schematic illustration of the Thermoclinching process based on [16]: (**a**) positioning of the pre-cut joining partners and heating-up of the joining zone, (**b**) permeating of the fibre reinforced structure with the tapered pin, (**c**) forming of the undercut, (**d**) releasing finished joint.

The Thermoclinching process is based on the combined features of thermoplastic riveting [18] and clinching with a pilot hole [5] forming a defined fibre-reinforced undercut. The tool concept is composed of a tapered pin and a rigid die with a movable annular anvil [16]. In a preliminary step, the composite material in the joining zone is cut in thickness direction and a pilot hole is drilled into the metal sheet. The joining process is illustrated in Figure 3. At first, the joining partners are positioned with the TPC sheet on the punch side and the metal sheet on the die side. Afterwards, the TPC sheet is locally heated above melting temperature to increase the deformability of the fibres inside the thermoplastic matrix (Figure 3a). The tools are also warmed up for keeping the formability of the composite while joining. In the next step, the pin moves downwards to reorient the fibres in thickness direction through the pilot hole in the metal joining partner (Figure 3b). At the end of the joining process, the passed through TPC material is compressed by the rigid die with an annular anvil to form the final undercut (Figure 3c). The forming of the joint takes less than 1 s. After consolidation of the TPC and releasing the pin and the die, the thermoclinched joint is finished.

### 2.2. Hotclinching

Hotclinching as a single-stage joining process for metal-TPC joints is an adaption of a conventional clinching process with a rigid die [17]. A two-part die consisting of a rigid sleeve and a spring-loaded anvil in combination with thermal support are used. The thermal support is provided via cartridge heaters and improves the formability of the TPC material. The process extends the applicability of clinching processes without pilot hole to TPC-metal joints, when the TPC is positioned on the die side.

**Figure 4:**
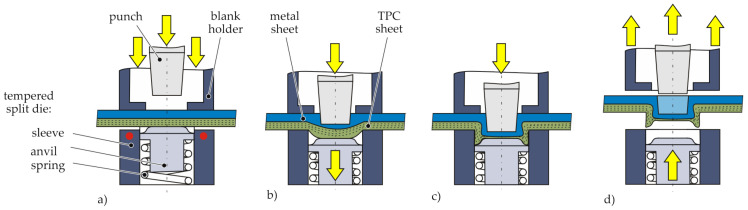
Schematic illustration of the Hotclinching process: (**a**) positioning, heating and fixation, (**b**) offsetting, (**c**) upsetting and flow pressing, (**d**) releasing finished joint.

At first, the joining partners are positioned between the heated split die and the blank holder (Figure 4a). Thereby, the TPC sheet is warmed up by contact heating by the tempered die. Afterwards the blank holder moves downwards followed by the punch. Due to the downward stroke of the punch a deformation and offsetting takes place (Figure 4b). During the offsetting, the spring-loaded anvil is pressed downwards, thus applying a counter-pressure to the joint. In this way, the neck area of the punch-sided sheet is stabilised, which improves the formability. As a result of the applied pressure, the punch-sided material flows in radial direction, whereby an undercut is created (Figure 4c). At the end of the process, the finished joint is released (Figure 4d).

### 2.3. Insert Clinching

**Figure 5:**
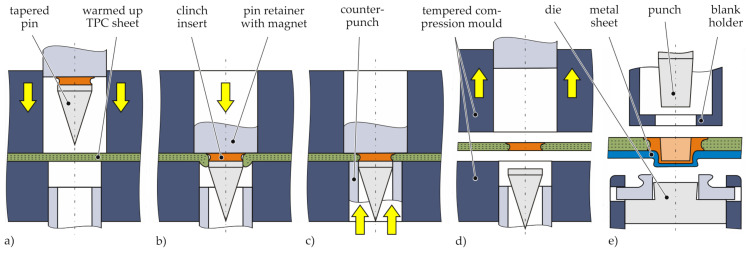
Schematic illustration of the Insert Clinching process: (**a**) compression mould closing, (**b**) movement of the pin tool, (**c**) recompressing the squeezed-out material by the counterpunch, (**d**) demoulding, and (**e**) subsequent standard clinching process with rigid or opening die.

Metal inserts can be embedded into TPC during part manufacturing process using the principle of moulding holes [19]. Thereby, the reinforcing fibres are not cut by punching or drilling, but shifted aside by a tapered pin tool in a plasticised state of the TPC [20]. Embedded inserts are suitable as an interface for joining TPC to metal using resistance element welding [21]. Furthermore, such inserts can be used as an interface for conventional clinching of hybrid joints. This novel approach called Insert Clinching is schematically illustrated in Figure 5. Steps (a) to (d) show the embedding process of the so called clinch insert during compression moulding of the TPC part and (e) illustrates the actual TPC-metal joint produced in a subsequent standard clinching process. First, the TPC sheet is warmed up above melting temperature of the matrix by an infrared heating device. Afterwards, the TPC sheet is quickly transferred into the open compression mould. Immediately after closing the tempered compression mould (Figure 5a), a tapered pin tool (consisting of pin retainer and tapered pin) is shifted forward, forming a hole by displacing the reinforcing fibres and the still molten matrix (Figure 5b). The two-parted pin tool contains a magnet to attach the clinch insert and the tapered pin to the pin retainer. Subsequently, the pin movement the squeezed-out material is recompressed by a ring shaped counterpunch, whereby the undercut of the clinch insert is filled with fibres and matrix material (Figure 5c). The embedding process, steps (b) and (c), takes less than 1 second. After solidification, the shaped TPC component with integrated clinch insert is demoulded (Figure 5d). Afterwards, the composite part can be joined with metallic components in a subsequent clinching process using standard tools (Figure 5e). Both rigid and opening dies are applicable. The TPC can be positioned punch-sided as well as at the die-side, which contributes to the flexibility in application. In the clinching process, the clinch insert and the metallic joining partner are deformed, while the TPC remains undeformed.

## 3. Materials and Methods

### 3.1. Material Specification

The materials used for process studies, analyses of the joining zones and mechanical testing are summarised in Table 1. Glass fibre reinforced polypropylene (GF-PP) and glass fibre reinforced polyamid 6 (GF-PA6) are typical materials for TPC applications with moderate thermal and mechanical requirements.

### 3.2. Equipment and Characteristic Dimensions

#### Thermoclinching

**Figure 6:**
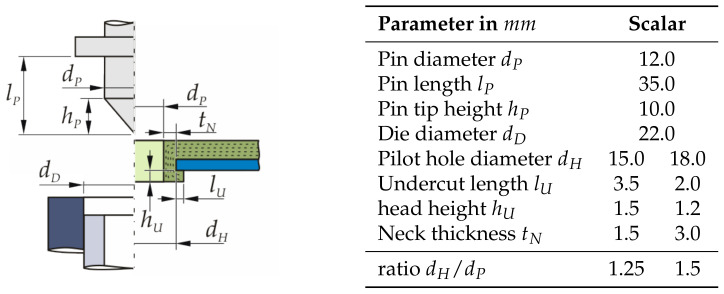
Schematic illustration of Thermoclinching joining zone and tools with parameters.

With regard to the occurring high deformation degree of the TPC specimen during Thermoclinching and in order to improve the local deformability of the textile structure, the TPC specimens in this work are locally cut crosswise and heated up to 200 °C in the area of the joining zone. The Thermoclinching process is performed on a developed joining system with tapered pin, rigid die, and annular anvil geometry (Figure 6). For the detailed investigation of the dependencies between process and design parameters and their effects on the qualitative formation of the Thermoclinching joining zone, the developed system is equipped with servo-pneumatic force and displacement control and an interchangeable tool set.

#### Hotclinching

**Figure 7:**
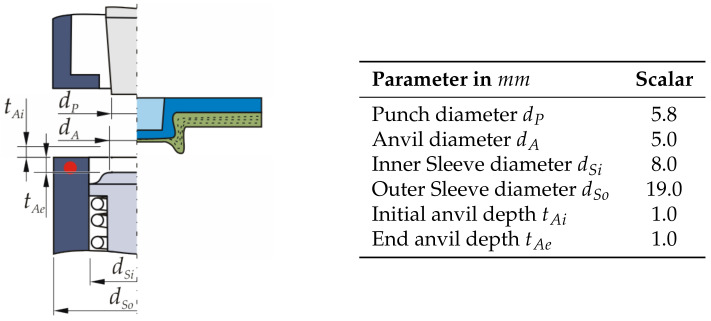
Schematic illustration of Hotclinching tools with parameters.

For the manufacturing of hotclinched joints, a clinching machine TOX${}^{®}$ MC-4.8 from TOX${}^{®}$ PRESSOTECHNIK GmbH & Co. (Weingarten, Germany) KG is used. The machine is a C-frame press with a stroke-controlled, pneumo-hydraulic driven joining cylinder. The limitation of the stroke is realised by the height adjustment of the die. To realise thermally assisted clinching by contact heating via heating cartridges, the die-sided tool holder is modified, as indicated in Figure 7. The TPC material is warmed up to 180 °C. Heating the TPC up to melting temperature can lead to a insufficient formation of an undercut caused by the reduced stiffness of the TPC in the bottom area. Therefore, the target temperature for the joining process has to be below the melting temperature of the thermoplastic matrix [17].

A schematic overview of the tool dimensions can be seen in Figure 7. The punch velocity during the process depends on the used material and clinching machine. In this investigation, it is 2 mm s^−1^. At the beginning of the process, the anvil protrudes above the rigid sleeve in the initial position $t_{Ai}$ and thus generates a counter-pressure on the joint. The anvil spring in the initial position is not pre-loaded and has a stiffness of 700 N/mm. In Figure 7, the end position of the anvil is illustrated.

#### Insert Clinching

**Figure 8:**
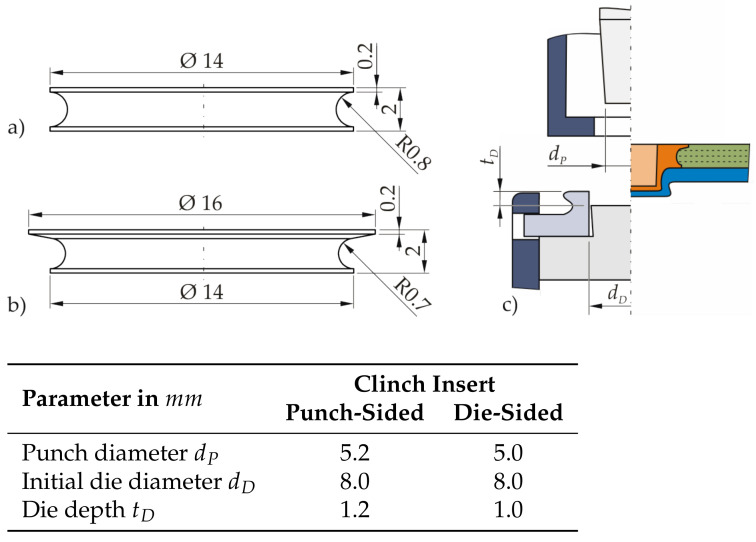
Characteristic dimensions of the used clinch inserts: (**a**) V1 and (**b**) V2 as well as (**c**) the clinching tool and joint.

Two different variants of clinch inserts are embedded in TPC specimens, both rotationally symmetric, see Figure 8. The height of the clinch inserts corresponds to the thickness of the TPC. Clinch insert V1 is furthermore axially symmetrical, which simplifies feeding in a potential industrial application. Clinch insert V2, on the other hand, has a larger head diameter on one side, which can be expected to result in higher joint strengths, especially under transverse tensile load.

To manufacture plane TPC specimens with integrated clinch inserts a developed pilot rig on laboratory scale consisting of an infrared heating device (210 °C) and a tempered steel mould (40 °C) with vertical flash face is applied. The pin tool is pneumatically actuated, such as the counterpunch.

For the clinching process conducted after insert embedding, a DFG 500/150 machine from ECKOLD GmbH & Co. KG is used. The C-frame stand machine with hydraulic drive has a stroke-controlled joining cylinder. The limitation of the stroke is realised via the height adjustment of the punch. Conventional clinching tools for metal joints are used, as can be seen in Figure 8c. The characteristic dimensions of the clinching tools are summarised in the table, shown in Figure 8. For the joining direction with the clinch insert positioned on the die side, the punch geometry is changed and the die depth is reduced, in order to increase the neck thickness of the thinner aluminium sheet positioned on the punch side.

### 3.3. Evaluation Methods

An analysis of the joining zone with imaging methods is essential to evaluate and categorise process phenomena and to qualitatively assess the clinch joints. Characteristic dimensions of a clinch joint are the undercut ($t_{U}$), the bottom thickness ($t_{B}$), and the neck thickness ($t_{N}$), which significantly affect the joint strength and thus the joint quality [22]. While the neck thickness predominantly has an impact on the shear strength of a clinch joint, the undercut mainly influences the cross-tensile and peeling strength [23]. For the geometrical analysis of a clinch element, photographs or micrographs of cross-sections are evaluated conventionally (cf. [9,24,25]). These methods have the disadvantage that the joining zone can only be evaluated in one section plane and thus three-dimensional phenomena, such as fibre reorientations, can hardly be evaluated. The three-dimensional material structure of a joining zone can be investigated by computed tomography (CT) analysis [19]. In addition to defects in the joining zone such as pores, delaminations, or cracks [26], the path of the reinforcing fibres can be analysed. In this work, both CT scans and micrographs are used to analyse the local material structure in the joining zone. For the analysis of the thermoclinched and hotclinched joints, an X-ray voltage of 80 kV, and a cathode current of 80 μA, a high resolution and high contrast imaging of the joining zone is possible.

**Figure 9:**
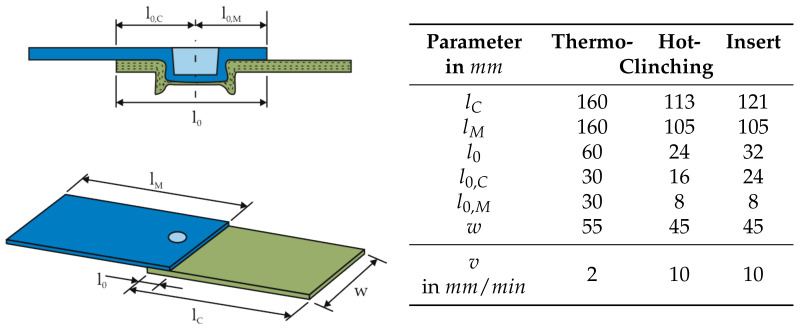
Specification and dimensions of the single-lap shear test specimens for the different clinching technologies.

To investigate the mechanical properties of clinched joints, various test methods are common. Often single-lap shear tests, transverse tensile load tests and peel tests at quasi-static load application are performed [27]. The most common method is the single-lap shear testing, for both metal and composite joints [28]. For this reason, single-lap shear tests are carried out for the assessment of the different clinching technologies in this paper. The geometries of the test specimens are shown in Figure 9. Due to the different geometric dimensions of the joining zones, the design of the test specimens and the testing velocities vary. Compared to Hotclinching and Insert Clinching, the joining zone is larger in Thermoclinching. Therefore the test specimens were designed in accordance with [29] (basis for [30]) in order to avoid influences of the edge areas on the failing behaviour. As for thermoclinched joints a failure of the TPC structure is to be expected, the testing velocity *v* was defined as 2 mm/min on basis of quasi-static tensile testing of TPC (cf. [31]). For Hotclinching and Insert Clinching, the dimensions of the specimen as well as the testing velocity *v* were set in accordance to [30]. In order to avoid an influence of the edge areas on the failure behaviour, the overlap length $l_{0,C}$ was adapted to the size of the joining zone.

## 4. Process Phenomena

In general, the main deformation of clinching processes is in thickness direction. In the Thermoclinching and Hotclinching processes, the forming of the TPC structure takes place during the actual clinching process. In contrast, in the Insert Clinching process, the TPC is formed during TPC component production and not during the clinching process. Especially for continuous fibre reinforced thermoplastics, the forming process changes the local material structure. In all three joining processes considered, the TPC is formed in a warmed up condition.

Three main phenomena can be observed during the joining process. The tool penetration and compaction of the joining zone by die or counterpunch lead to a change of the fibre paths, including fibre reorientation both in thickness direction and in laminate plane direction. If the penetration and the stroke of the tools lead to an exceeding of the elastic properties of the fibres, fibre failure occurs. The failure modes of the fibres vary between bending or tension in fibre direction.

The complex material structure resulting from those process phenomena has a significant influence on the load bearing behaviour, as could be shown by the example of warm-embedded inserts [20]. The resultant material structure depends on the geometry of the tools, the process parameters, and the textile architecture.

### 4.1. Thermoclinching

**Figure 10:**
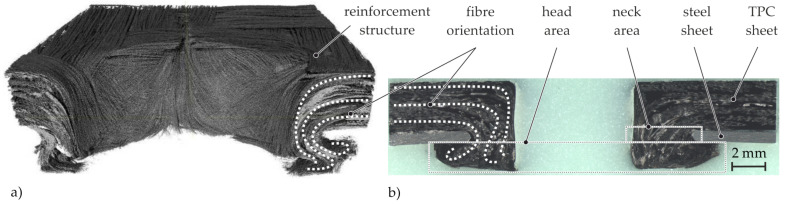
(**a**) CT analysis of the TPC of a thermoclinched joint and (**b**) micrograph analysis of a Thermoclinching joint.

For the understanding of the local material structure of thermoclinched joints both micrographic and CT analysis are used. The micrographic analysis shows that parts of the textile reinforcement are specifically relocated to the neck and head area of the final joint (Figure 10b). This relocation of the textile reinforcement considerably contributes to the load carrying capacity of the joints [16].

For a more detailed understanding of the occurring principle deformation characteristics in the thermoclinched joining zone, CT analyses are performed (Figure 10a). It can be seen that the reorientation of the reinforcing fibres is accompanied by various deformation phenomena. Thus, there is a relocation of the fibres into the form-closed head area including fibre reorientation both in thickness direction and in plane direction of the joints head. Thereby, splaying of the roving ends can be observed. In order to analyse the occurring deformation behaviour during the joining process, more in depth analyses were carried out [16]. The studies show that a stepwise CT analysis serves as an adequate method to identify and describe the principle deformation characteristics during the Thermoclinching process. Furthermore it was observed that the deformation characteristics, and therefore the joint quality, is significantly dependent on a wide range of parameters such as tool geometry and joint dimensions, as well as material of the joining partners and process parameters [32].

### 4.2. Hotclinching

**Figure 11:**
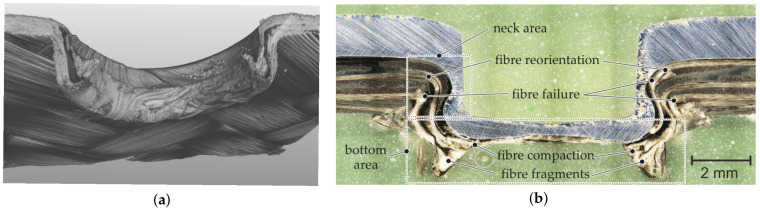
(**a**) CT analysis of the TPC of a Hotclinching joint and (**b**) micrograph of a Hotclinching joint (DC04/GF-PA6).

In case of the Hotclinching process, all three major phenomena can be observed. An example of the resultant material structure of a Hotclinching joint can be seen in the CT analysis in Figure 11a, and a micrograph in Figure 11b. In the micrograph fibre reorientations in thickness direction can be seen, mainly occurring in the heating zone of the die. In the neck area of the joint, the fibres are bended in motion direction of the punch. As can be seen, bended fibres next to the formed undercut failed, which means that the critical stress in this area was exceeded. Especially in the bottom area of the joint and in the ring groove of the anvil excessive fibre failure and radial movement of the fibre fragments occurs. The material flow of the die-sided joining partner favours the formation of an undercut [11]. The flow pressing of the metal joining partner and resultant undercut forming can be used to explain the described fibre failure in the neck area of the joint. In cause of the flow pressing and the displacement of the composite in the bottom area, compaction phenomena in the area of the ring channel can be observed, as the bottom thickness of the TPC is significantly reduced.

Regarding the quality characteristics of the hotclinched joints, it can be stated that joints for the material combination DC04–TPC have an average undercut of $t_{U}$ = 0.39 mm and a neck thickness of $t_{N}$ = 0.66 mm. For the combination EN AW-6016 T4–TPC, the average undercut ($t_{U}$ = 0.36 mm) and a neck thickness ($t_{N}$ = 0.62 mm) are slightly lower.

### 4.3. Insert Clinching

**Figure 12:**
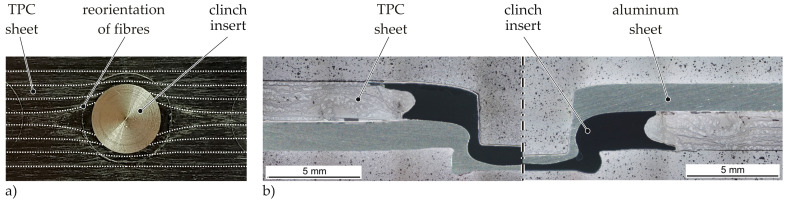
(**a**) Photograph of a TPC specimen with embedded clinch insert V1, (**b**) micrographs of clinch joints with punch- and die-sided clinch insert V2.

The laminate surface of TPC specimen with embedded clinch inserts is analysed visually by means of photographs before clinching (Figure 12a). In addition, microscopic examinations of cross sections of clinched joints are carried out for the analysis of the Insert Clinching process (Figure 12b). During embedding of the clinch insert, fibres and plasticised matrix are initially displaced by the pin movement both laterally in the laminate plane (cf. Figure 12a) and in the thickness direction (cf. Figure 12b). The material displaced in thickness direction is pressed back into the laminate plane afterwards by the counterpunch. A complete filling of the undercut of the clinch insert with reinforcing fibres and matrix can be achieved during the embedding process as a result of the compression by the counterpunch (cf. Figure 12b). This is possible due to the high temperature in the forming process, which leads to high movability of the reinforcing fibres and the thermoplastic matrix. Thus, the embedding process results in a local complex material structure with an inhomogeneous three-dimensional fibre orientation and locally varying fibre content. This is in accordance with the analyses in [20]. Fibre failure could not be determined.

During the actual clinching process, there is no significant change in the laminate structure, as the clinch element is formed between the clinch insert and the steel sheet. For this reason, standard quality characteristics such as neck and bottom thickness as well as the undercut can be used for the evaluation of the clinch element. Joints with the clinch insert positioned on the punch side show an average undercut of $t_{U}$ = 0.22 mm and a neck thickness of $t_{N}$ = 0.48 mm. For the opposite joining direction (insert die-sided), an average undercut of $t_{U}$ = 0.18 mm and a neck thickness of $t_{N}$ = 0.29 mm are achieved. The significant variation, especially concerning the neck thickness, can be explained by the different thickness and strength ratios of the insert and the aluminium sheet. When the thinner aluminium sheet is positioned on the punch side, the material has to be drawn deeper in order to form an undercut in the insert. In this case the neck area is elongated and thinned. Nevertheless it can be stated, that for both joining directions appropriate joints can be achieved.

## 5. Load Bearing Behaviour

For the application-oriented qualification of the three joining methods, discussed in this work, single-lap shear tests of the previously presented configurations are carried out under quasi-static load application. Besides the maximal load, special attention is paid to the respective failure behaviour of the composite materials. Table 2 summarises the main characteristics of the investigated joints.

### 5.1. Thermoclinching

**Figure 13:**
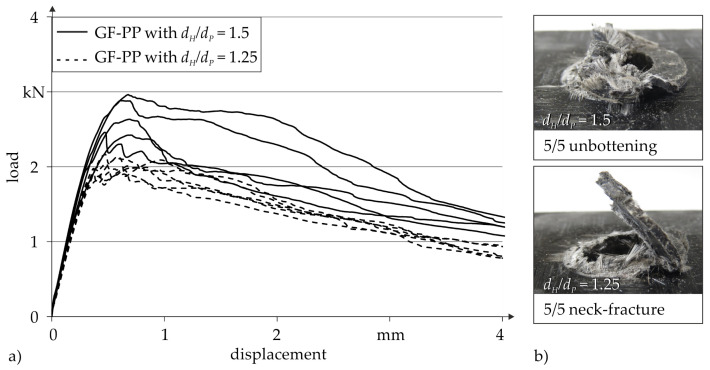
Thermoclinching: Results of single-lap shear tests under quasi-static load for thermoclinched joints with different pilot hole to pin diameter ratios $d_{H}$/$d_{P}$: (**a**) load-displacement curves, (**b**) characteristic failure behaviour.

Figure 13a shows the load displacement curves resulting from single-lap shear tests on thermoclinched specimens with different pilot hole to pin diameter ratios $d_{H}$/$d_{P}$.

It can be seen that the joints with $d_{H}$/$d_{P}$ = 1.5 have higher load-bearing capacities than those with $d_{H}$/$d_{P}$ = 1.25. This repatriates from the resulting joining zone geometry, whereas $d_{H}$/$d_{P}$ = 1.5 leads to an increased neck thickness $t_{N}$ and a reduced head height $h_{U}$. In difference, $d_{H}$/$d_{P}$ = 1.25 results in a low neck thickness $t_{N}$ and corresponding high head height $h_{U}$, showing a reproducible load-deformation behaviour with average maximum forces of 2.0 kN. Thus, the different $d_{H}$/$d_{P}$-ratios also have an impact on the failure behaviour, and two different failure modes can be observed (Figure 13b). With higher material concentration in load direction the increasing neck thickness of $d_{H}$/$d_{P}$ = 1.5 induces an unbuttoning of the joint, pulling the head through the pilot hole of the steel sheet. In contrast, joints with low neck thickness ($d_{H}$/$d_{P}$ = 1.25) show a shearing failure. Thereby, the fibres in the neck area of the joint are cut by the steel sheet, separating the head from the rest of the joint. Both joint geometries show a successively decreasing load capacity, excluding a total failure behaviour of the joints.

### 5.2. Hotclinching

**Figure 14:**
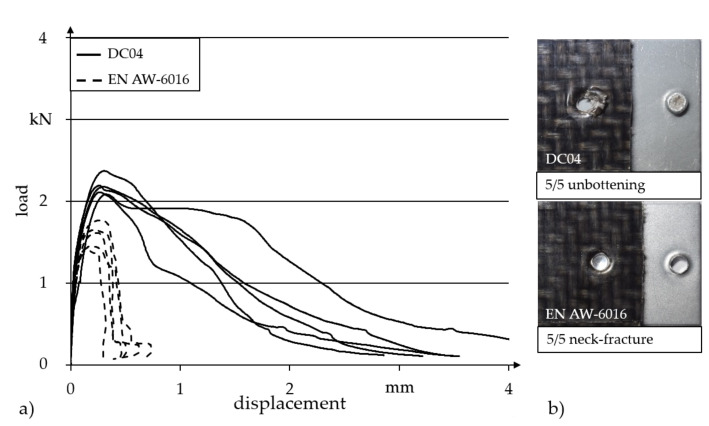
Hotclinching: Results of single-lap shear tests under quasi-static load for DC04 and EN AW-6016 as metallic joining partner: (**a**) load-displacement curves, (**b**) characteristic failure behaviour.

Figure 14a shows the load displacement curves for conducted single-lap shear tests on hotclinched specimens. For the investigations, both the steel material DC04 and an aluminum sheet EN AW-6016 T4 were used as metal joining partners.

It can be seen that the DC04-TPC joints have significantly higher load-bearing capacities (average 2.2 kN) than those joined with the aluminium sheet material (average 1.6 kN). In addition, the failure displacement is noticeably higher, which results in a significant increase in energy absorption of the joint. The lower load capacity and fracture displacement of the Al-TPC joints can be explained by the failure characteristic of the joints. It can be seen in Figure 14b that the failure behaviour of the DC04-TPC joints is characterised by a localised bearing failure with subsequent buttoning out. In the case of the material combination Al-TPC, the joints failed by neck fractures of the aluminium sheet which leads to the abrupt load drop after exceeding the maximal load. This can be explained due to the lower strength of the aluminium material compared to the steel sheet DC04, as the neck thicknesses for both material combinations were comparable.

### 5.3. Insert Clinching

**Figure 15:**
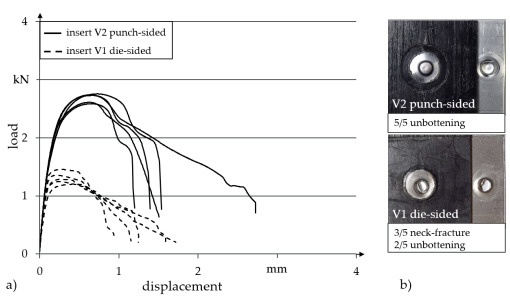
Insert Clinching: Results of single-lap shear tests under quasi-static load for clinch inserts in punch-sided and die-sided orientation: (**a**) load-displacement curves, (**b**) characteristic failure behaviour.

Figure 15 shows the results of single-lap shear tests of joints with the two clinch inserts described. Both joining directions were considered. For the joining direction with the insert positioned on the punch side, the insert V2 with an expanded head diameter is used, as higher maximum shear loads were to be expected. In contrast, clinch insert V1 (axially symmetric) is positioned die-sided.

Clinched joints with insert V1 positioned on the die side provide a maximum shear load of 1.3 kN on average. The average maximum shear load of a specimen with clinch insert V2 positioned on the punch side is 2.7 kN. The different load-bearing capacities go along with different failure behaviours of the specimens. Positioning the clinch insert on the die side leads to neck fractures of the punch-sided metal joining partner analogous to hotclinched joints with the aluminium joining partner. Furthermore, in this configuration, the punch-sided aluminium sheet (1.5 mm) is thinner compared to the die-sided insert (2.0 mm), which promotes lower neck thicknesses. When the clinch insert is arranged on the punch side, an unbuttoning of the insert out of the aluminium sheet occurs due to the high neck thickness of the steel insert. With regard to the failure behaviour of the joints, it should be emphasised that in each case, a separation of the clinch joint between the two metal parts is the cause of the failure, and the joint between the insert and the surrounding TPC remains intact in each case. It can be stated that the performance of the clinched joints for these configurations are fully achieved.

## 6. Discussion

In the present paper, three different novel joining technologies are presented and compared with a focus on process cycle, resultant material structure, and load bearing capacity. For a comparison of the achieved joint strengths with the state of the art, the obtained shear loads of the technologies discussed in the introduction (cf. Figure 2) are given in Table 3 along with the used materials and sheet thicknesses. It can be seen that in most cases, aluminium sheets with thicknesses of 1.4 mm to 3.0 mm as metallic joining partners are considered. Regarding the composites, a wide range of reinforcement and matrix combinations are used in thicknesses from 1.0 mm to 4.0 mm. For the different joining processes and materials used, the maximum shear loads (1.3 kNto 3.3 kN) of the joints are in comparable magnitudes. Due to the load-bearing capacities achieved, these methods are suitable for non-structural applications or in combination with adhesive bonding, a fixation for handling can be realised until the adhesive is cured (hybrid joint).

For the processes examined, it can be concluded that a generalised statement about which method is best suited for an application cannot be given exclusively by considering the load-bearing capacities. Rather, the normalised shear strength should be regarded. However, for a valid comparison, aspects such as materials, sheet thicknesses, joining tool dimensions, and the geometry of the joining zone also have to be considered and summarized, for instance, in the form of a coefficient. Defining such a coefficient in detail, however, is hardly practicable because of the many specific aspects that have to be included.

For this reason, unique characteristics of the three processes are discussed below in relation to application-relevant criteria (Table 4), as well as the observed process phenomena. Thermoclinching is a joining technology for thick TPC and thin metal sheets. In comparison to [8] (Table 3a) high shear loads of 2.6 kN for a metal sheet thickness of 1.0 mm are achieved. Since the metallic joining partner is not deformed in the clinching process, even metals with low ductility, such as ultra-high-strength steels (e.g., 22MnB5), can be joined. As both sheets have a local preparation, high positioning accuracies of the sheets to each other are required (Table 4). For this reason, Thermoclinching was improved regarding a robust and reproducible joining process [32]. Thereby, the cutting, heating, and joining processes are combined in an inline-Thermoclinching process to increase the process capability and reduce cycle times. This also reduces the requirements regarding positioning accuracy.

Hotclinching as a single-stage joining process that has no special requirements regarding the relative positioning of the sheets. In comparison to Thermoclinching, the process cycle including heating of the TPC is significantly faster (Table 4). The maximum shear load of 2.2 kN is in the same range as [15] (Table 3e). In contrast to Lambiase et al. [11], less fibre failure can be observed in the joining zone. Another advantage of the Hotclinching process is the suitability for hybrid joints.

The Insert Clinching shows comparatively high shear loads regarding metal sheets of 1.5 mm thickness. One of the main advantages is the use of conventional clinching tools. This allows the integration of composites in existing assembly lines without modification of the joining tools or process chains. The process is more suitable for thin TPC sheets (<3.0 mm), as thicker clinch inserts are difficult to join without extending the tool geometries. In addition, the clinchability is not limited by the properties of the TPC. In principal, all common clinchable metal–metal combinations can be joined, as inserts of different materials can be embedded. In contrast to the other clinching methods, both joining directions are applicable. Compared to Hotclinching, there are requirements regarding the positioning accuracy. The TPC sheet and the metal sheet do not have to be positioned exactly in relation to each other, but the clinching tool must hit the position of the clinch insert. Further investigations have shown that up to a lateral deviation between the tools and the insert of 1.5 mm applicable joints can be achieved.

Regarding the process phenomena during joining, it can be stated that thermal support is used in all processes in order to increase the formability of the composite during joining (Thermoclinching, Hotclinching) or to embed a clinch insert (Insert Clinching). A change in the local material structure of the TPC is common to all methods, but the observed phenomena occur to varying degrees. In the Thermoclinching process an initial fibre failure is induced intentionally to enable the fibre bending for the joining process. For Hotclinching fibre failure occurs during the deformation process because the process induced fibre tension exceeds the fibre strength. In contrast for Insert Clinching, the fibres are reoriented during the embedding process of the clinch insert without fibre failure, while the TPC is not affected by the subsequent clinching process. Since all three processes operate with (nearly) same tool velocities, it can be concluded that the strain rates and strain rate effects of the material are at the same level. Therefore, the different temperature levels and degrees of deformation are most important to explain the process phenomena.

## 7. Conclusions

The present paper compares three different clinching technologies for TPC-metal joints regarding the required process steps and process parameters for a specific material combination. In micrographs and CT analysis, it could be shown that a thermal support during deformation process of the TPC leads to less fibre damage and enables a better fibre reorientation. As a result, for clinching TPC, thermal support is suggested during the deformation process.

In order to evaluate the mechanical properties of the joints single-lap-shear test were carried out for all three technologies. It is shown that the achievable load-bearing capacities are in the same order of magnitude as for the clinching technologies described in the literature, while the considered processes reduce fibre damage and do not cause a contamination of the clinching tools. However, further criteria such as process design, preparation effort, materials, and sheet thicknesses must be taken into consideration for the selection of an application-appropriate clinching technology. For instance, Thermoclinching offers the possibility to join ultra-high-strength steels or thick TPC sheets. Hotclinching enables joining without any preparation step or requirements for positioning accuracy of the joining partners. The novel Insert Clinching technology allows standard metal clinching with conventional tools.

For an advanced comprehensive analysis of the load bearing behaviour and comparison of the joining technologies in further investigations, it is recommended to characterise the joints by defined mixed-mode ratios and pure-mode tests, like those shown in [33].

## Figures and Tables

**Table 1:** Specification of the utilised materials.

		Thermoclinching	Hotclinching	Insert Clinching
TPC	Material	GF-PP	GF-PA6	GF-PP
	Configuration	twill fabric [(0${}^{\circ }$/90${}^{\circ }$)_4_]_s_	twill fabric [(0${}^{\circ }$/90${}^{\circ }$)_2_]_s_	UD [(0${}^{\circ }$/90${}^{\circ }$)_4_]_s_
	Fibre volume content	35%	47%	45%
	Thickness	4 mm	2 mm	2 mm
Metal	Material	structural steel	steel DC04, EN AW-6016 T4	EN AW-6016 T4
	Thickness	1 mm	1.5 mm	1.5 mm
	Auxiliary insert	-	-	steel S235JR

**Table 2:** Characteristics of the investigated joints.

Process	Thermoclinching Ratio $d_{H}/d_{P}$		Hotclinching Joining Partner		Insert Clinching Insert Position	
	1.25	1.5	Steel	Aluminum	Punch-Sided	Die-Sided
Neck thickness $t_{N}$ in mm	1.5	3.0	0.66 ± 0.02	0.62 ± 0.03	0.48 ± 0.02	0.29 ± 0.02
Undercut $t_{U}$ in mm	3.5	2.0	0.39 ± 0.01	0.36 ± 0.01	0.22 ± 0.02	0.18 ± 0.01
Shear load in kN	2.0 ± 0.1	2.9 ± 0.1	2.2 ± 0.1	1.6 ± 0.1	2.7 ± 0.1	1.3 ± 0.1
Failure mode	neck fracture	unbuttoning	unbuttoning	neck fracture	unbuttoning	neck fracture

**Table 3:** Comparison of the maximum shear loads.

Technology	Material	Thickness in mm	Shear Load in kN
(**a**) Improved hole clinching [8]	Al/ thermoset CFRP	1.4/1.0	2.6
(**b**) Friction assisted clinching [9]	Al/thermoset CFRP	2.0/2.0	up to 1.3
(**c**) Thermoclinching [16]	steel/GF-PP	1.0/4.0	2.6
(**d**) Clinching-hole [6]	Al/thermoset CFRP	2.4/1.0–1.6	up to 3.3
(**d**) Clinching-hole free [11]	Al (AA6082-T6)/thermoset GFRP	2.0/2.0	1.9
	Al (AA5086)/thermoset GFRP	2.0 & 3.0/2.0	up to 2.0
(**d**) Clinching-hole free [12]	Al/thermoset CFRP	2.0/1.4	2.7
(**e**) Flow Drill Joining [15]	steel/CF-PA6	1.5/1.5	2.3
	steel/GF-PP	1.5/1.0	1.3
(**f**) Two-Step Clinching [13]	Al/thermoset CFRP	3.0/1.4	2.4
(**g**) Hotclinching [17]	steel/GF-PA6	1.5/2.0	2.2
	Al/GF-PA6	1.5/2.0	1.6
(**h**) Preheated Clinching [14]	Al/CF-TPC	1.6/1.6	2.5
(**i**) Insert Clinching	Al/GF-PP	1.5/2.0	up to 2.7

**Table 4:** Comparison of the three clinching technologies’ properties.

Criterion	Thermoclinching	Hotclinching	Insert Clinching
Joining direction	TPC to metal	metal to TPC	both
Clinch	30 s (heating)	16 s (heating)	<5 s
process time	+1 s (joining)	+3 s (joining)	(standard metal
	+30 s (consolidation)		clinching)
Complexity of the clinch tool	tapered pin/rigid die & annular anvil	conventional punch/spring-loaded die with heat source	conventional tools
Positioning accuracy	±1.0 m m	no requirements	±1.5 m m

## Data Availability

The data presented in this study are available on request from the corresponding author.

## Abbreviations

Al	Aluminium
Al-CFRP	Aluminum-carbon fibre reinforced plastic
Al-GFRP	Aluminum-glass fibre reinforced plastic
CFRP	Carbon fibre reinforced plastic
CT	Computed tomography
GF-PP	glass fibre reinforced polypropylene
GF-PA6	glass fibre reinforced polyamid 6
TPC	Thermoplastic composites
